# Supplementary material for: Chronic Chemogenetic Stimulation of the Nucleus Accumbens Produces Lasting Reductions in Binge Drinking and Ameliorates Alcohol-Related Morphological and Transcriptional Changes
Source: Brain Sci. 2020 Feb 18;10(2):109. doi: 10.3390/brainsci10020109 (PMC7071376; doi:10.3390/brainsci10020109)
Supplement: Supplementary file 1 [file brainsci-10-00109-s001.zip › brainsci-683721/Supplemental Materials_R1.docx]

**Supplementary Gene Expression Results and Discussion**

Reductions in ethanol consumption observed during the washout period of the DID paradigm suggest lasting, plastic changes as a result of chronic neuronal stimulation in mice expressing hM3Dq. We first identified DEGs for each treatment group as compared with the control group [H2O(VEH)]. Top genes resulting from DE are shown in Figure S1. Volcano plots for three group comparisons highlight the top 20 genes with the lowest p-values within each comparison. All comparisons shown, are presented relative to the H2O(VEH) control group. (A) DEGs in EtOH(VEH) group, (B) DEGs in EtOH(CNO) group, and (C) DEGs in H2O(CNO) group.

Next, we employed transcriptomic analyses to identify genes associated with binge-like drinking that were ameliorated by CNO. To do this, we identify DEGs effected by ethanol that were rescued by CNO, a Euclidean distance measure clustering was used. The top 47 genes with greatest variability (pairwise Euclidean distance > 1.5 standard deviations from mean) in expression patterns unique to EtOH (VEH) are illustrated. Gene lists and expression values are listed in Supplementary Table S2. Here, a majority of these DEGs were significantly enriched in four categories: positive regulation of immune responses, general immune response-regulating signaling pathways, immune response-activating signal transduction and activation of immune responses. This indicated that gene expression changes significantly perturbed by binge-drinking, disproportionately impact expression of genes in neuroimmune pathway regulation.


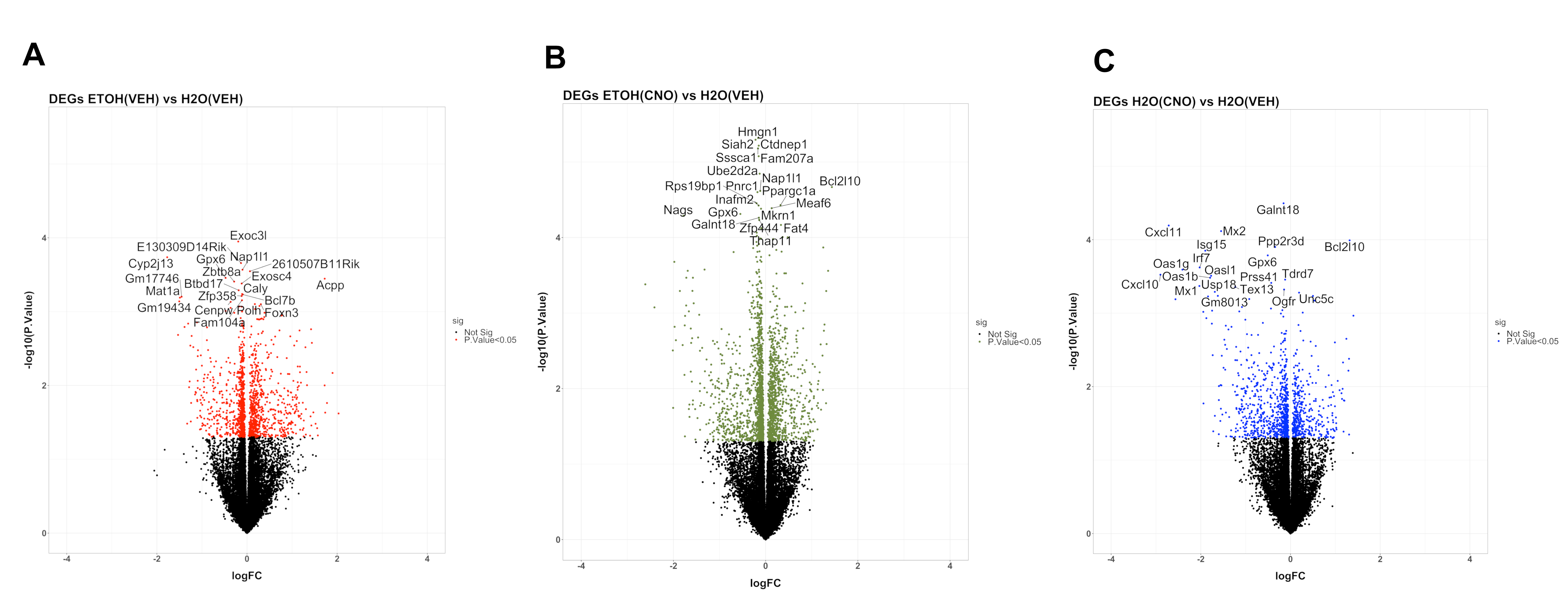


Supplementary Figure 1. Top genes resulting from DE. Volcano plots for three group comparisons highlight the top 20 genes with the lowest p-values within each comparison. All comparisons shown, are presented relative to the H2O(VEH) control group. (A) DEGs in EtOH(VEH) group, (B) DEGs in EtOH(CNO) group, and (C) DEGs in H2O(CNO) group.

Lastly, weighted gene covariance network analysis (WGCNA) was used to construct a scale free network and identify modules. Genes with a high degree of network connectivity (hubs) that correlated with binge-like drinking were used to identify candidate therapeutic targets. Gene overlap identified from DE implicated changes in gene expression that were either treatment and/or fluid type specific. The 688 genes changed solely in the group EtOH (VEH) indicate genes whose expression was perturbed exclusively by chronic binge-like alcohol drinking. This is the first time that NAc transcriptomic changes related exclusively to either chronic binge-drinking or chronic CNO stimulation of hM3Dq have been identified. For EtOH (CNO), 1,431 genes were found to be related exclusively to those genes ameliorated by CNO treatment. Further, 612 genes were identified in the H2O (CNO) group and represented genes that were related exclusively to chronic CNO-induced activation of hM3Dq.


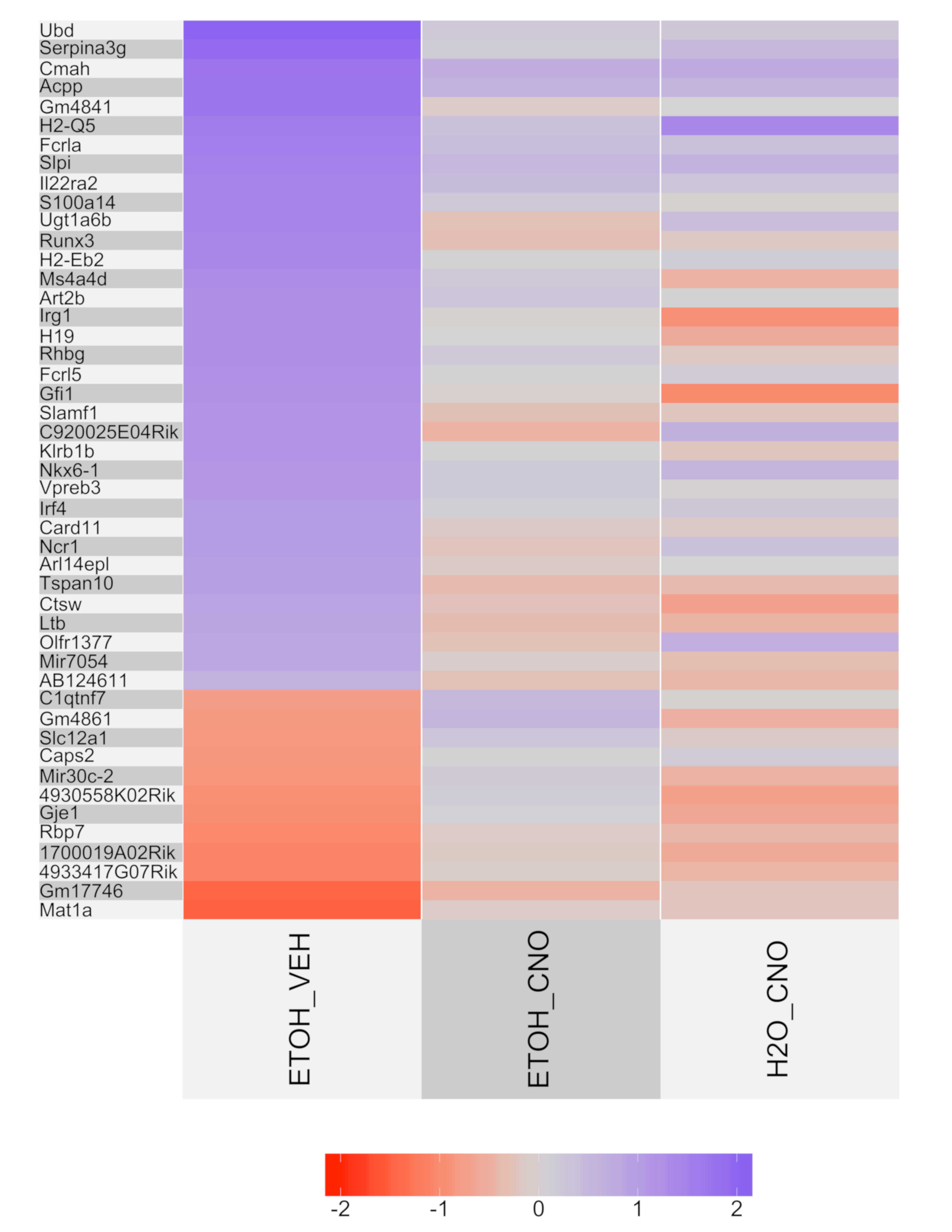


*Supplementary Figure 2. Gene expression changes produced by chronic binge drinking and stimulation of Gq signaling in the NAc. The top 47 genes with greatest variability (pairwise Euclidean distance > 1.5 standard deviations from mean) in expression patterns unique to EtOH (VEH) are illustrated. Gene lists and expression values are listed in Supplementary Table S2.*

WGCNA identified 13 distinct gene expression modules, where each module contains genes with correlated patterns of gene expression representative of shared biological functions and pathways. The cluster dendrogram is illustrated in Figure S3A. Gene dissimilarity based on topological overlap (height) is assigned to module color. A table containing genes with the highest degree of correlation (hubs) in each module is presented in Figure S3B. Figure S3B also shows the relative module size, as well as the hub gene description and the number of unique DE genes in each module (detailed gene lists for each module are located in Supplementary Table S4). In Figure S3C, the module eigengene (ME) for each module was calculated and clustered to illustrate inter-modular relationships. Module dissimilarity based on eigengene topological overlap is expressed on the y-axis. Increased height corresponds to lower similarity between modules, while decreased height corresponds to increased similarity.

Figure S3D illustrates the relationship of each module to each group. The ME-trait correlation measures the strength and direction of association between the module and the group trait. Relating group traits with a ME is descriptive of the general expression of all genes within that module, where negative values are associated with lower expression (red) for a group and positive values are associated with an increase in expression (blue) for that group. The expression value is indicated in each cell, with the associated p-value directly below in parentheses. Only the brown module was found to have a significantly, positive relationship with the EtOH(VEH) group (p <0.01). Although, this result may be predominantly driven by expression from a few mice. Conversely, three modules were significantly related to the EtOH (CNO) and two to H2O(VEH). The grey60 module was negatively correlated in EtOH(CNO) (p <0.05). The grey module (which is different than the grey60 module) represents unassigned genes, and was significantly correlated to both EtOH(CNO) (positive, p< 0.05) and H2O(VEH) (negative, p <0.01). The light cyan module was uniquely positively correlated to EtOH(CNO) (p <0.05), while the black module was uniquely positively correlated to H2O(VEH) (p <0.05). Focusing on the black module, the expression distribution of this module is not only conserved across groups, but it is also significantly, differentially correlated with the control (H2O(VEH)) and EtOH(VEH). This result implicates the direct susceptibility of gene expression in the black module to binge-like drinking and highlights it as a point of interest.

We created a consensus network using WGCNA and identified that clustered modules were correlated to treatment and fluid groups. Here, treatment groups were treated as traits. EtOH (VEH) was considered to be the binge drinking trait without treatment, EtOH (CNO) binge drinking with treatment and H2O (CNO) treatment with CNO alone. Modules (containing groups of genes) significantly correlated to treatments were identified as being driven by a particular trait. In this way, most of the genes in a given module significantly correlated to a certain trait, would also exhibit a correlation with the trait of the same sign as the eigengene. The eigengene-trait correlation measured the strength and direction of association between the module (the representative profile) and the trait. Thus, if this was positive (or negative), it implied that the trait increased (or decreased) with increasing eigengene "expression". For example, the brown module was significantly correlated to the EtOH (VEH) trait [but not the EtOH (CNO) or H2O (CNO) traits], thus binge drinking was being driven positively by the majority of genes in the brown module. Interestingly, there were no modules significantly correlated with CNO. This implied that 1mg/kg/day CNO had no significant lasting impact on correlated gene changes and thus may have little to no significant effect on biological processes. This result is further supported previously by groups such as Jendryka et al., (2019) which showed that off target effects of CNO are minimal at low doses (Jendryka et al., 2019). Further, we used our data set to query the LINCS L1000 database (Koleti et al., 2018) and found low connectivity with clozapine, suggesting there was not a significant contribution from back metabolism of CNO to clozapine.

Hdac4 was identified in the black module. This module was found to be significantly negatively correlated to the H2O (VEH) trait, while being positively correlated to EtOH (VEH) though not significantly. By nature, trait and module correlation is directly related to gene expression. Thus, this inverse correlation suggests that changes in gene expression are directly related to a difference in trait correlation. This implies that if Hdac4 expression were changed, it (as a regulatory gene) would induce a cascade of expression changes (in this particular differentially correlated module) that could ameliorate changes induced by binge drinking. The black module Hub is the gene Usp9x (Ubiquitin Specific Peptidase 9 X-linked or Deubiquitinase). Deubiquitinase is involved both in the processing of ubiquitin precursors and of ubiquitinated proteins. Thus, it plays an important regulatory role at the level of protein turnover by preventing degradation of proteins through the removal of conjugated ubiquitin (UniProt Consortium, 2008). EnrichR GO analysis for transcription factors in this module revealed Hdac4 and Usp9x among other genes were highly over represented for the transcription factors Ctnnb1 and Jun. Both transcription factors regulate many genes with numerous, complex roles in cellular processes (Kuleshov et al., 2016). Interestingly, although Hdac4 is not the primary hub gene within the black module, it is modulated by the same transcription factors as the hub gene and participates in a similar biological pathway. Thus, we infer that changing the expression of either of these genes would impact the overall correlation of the module they are found in to a particular trait. This result presents a significant finding for further pharmacological targeting of genes that participate in this module in order to ameliorate gene expression changes that are caused by binge drinking. These findings have resulted in the identification of Hdac4 and other genes, such as Usp9x, in this black module as important for amelioration of gene expression related to alcohol use disorders.


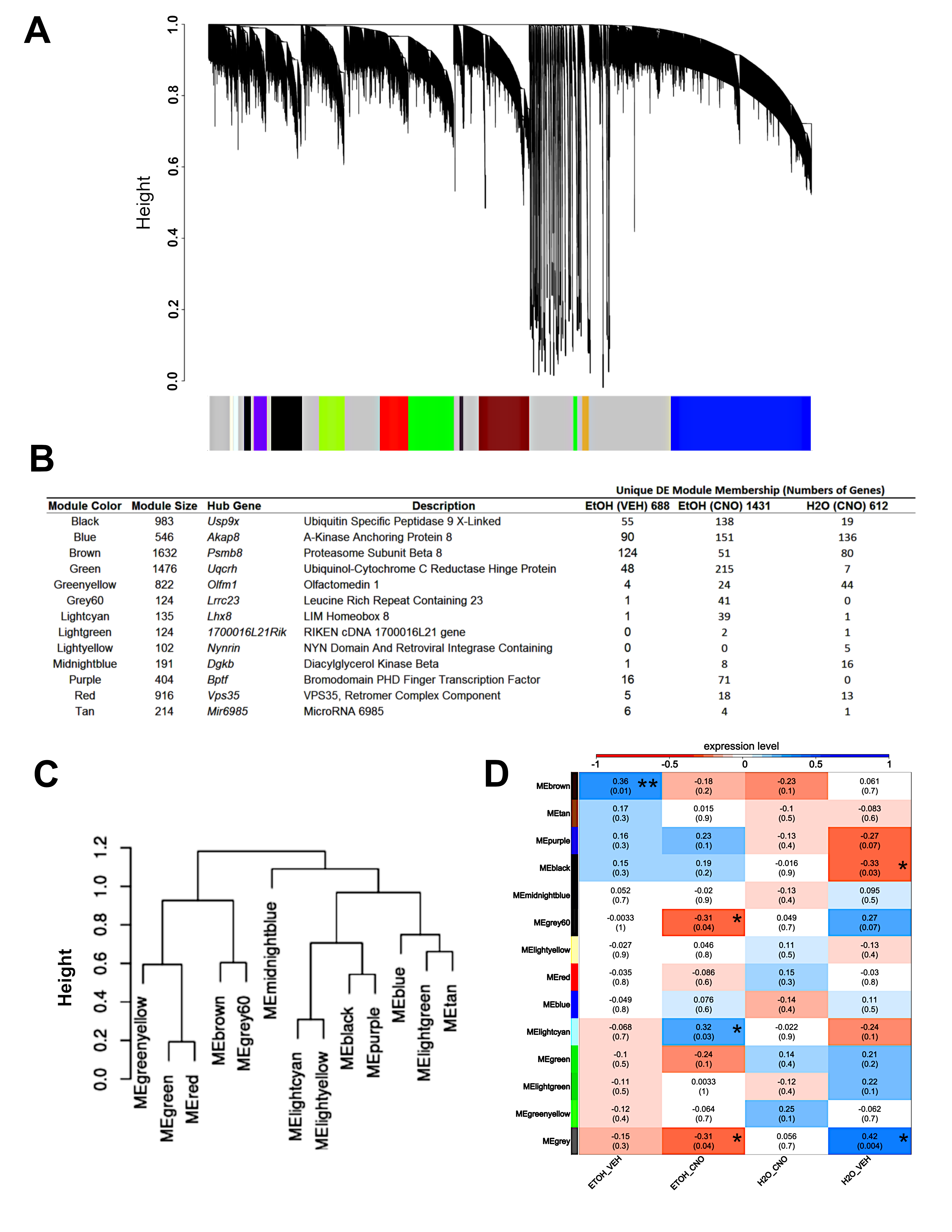


Supplementary Figure 3. Effects of DREADDs and Drinking on Gene Networks. (A) Gene dendrogram showing the co-expression modules defined by labeled colors. Gene dis-similarity is shown as height. (B) Table describing module characteristics, hub genes and their descriptors, and module membership of unique DEGs identified in Figure 3. (C) Eigengene dendogram showing the correlation of modules as labeled colors corresponding the (B). The dis-similarity of modules is expressed as height. (D) Matrix with the Module-Trait Relationships with expression values (and corresponding p-values) between the detected modules (rows) and fluid type and treatment traits (columns). Blue Module-Trait relationships indicate a strong upregulation, while red indicates a strong down regulation (and white indicates little or no change). * p < 0.05, ** p <0.01.
